# Association of Flavonifractor plautii, a Flavonoid-Degrading Bacterium, with the Gut Microbiome of Colorectal Cancer Patients in India

**DOI:** 10.1128/mSystems.00438-19

**Published:** 2019-11-12

**Authors:** Ankit Gupta, Darshan B. Dhakan, Abhijit Maji, Rituja Saxena, Vishnu Prasoodanan P.K., Shruti Mahajan, Joby Pulikkan, Jacob Kurian, Andres M. Gomez, Joy Scaria, Katherine R. Amato, Ashok K. Sharma, Vineet K. Sharma

**Affiliations:** a Metagenomics and Systems Biology Group, Department of Biological Sciences, Indian Institute of Science Education and Research Bhopal, Bhopal, India; b Department of Genomic Science, Central University of Kerala, Kasargod, India; c Department of Oncology, Amala Institute of Medical Sciences, Thrissur, India; d Department of Animal Science, Department of Food Science and Nutrition, University of Minnesota, Saint Paul, Minnesota, USA; e Animal Disease Research & Diagnostic Laboratory, South Dakota State University, Brookings, South Dakota, USA; f Department of Anthropology, Northwestern University, Evanston, Illinois, USA; Vanderbilt University

**Keywords:** colorectal cancer, gut microbiome, *Flavonifractor plautii*, biomarkers

## Abstract

Recently, dysbiosis in the human gut microbiome and shifts in the relative abundances of several bacterial species have been recognized as important factors in colorectal cancer (CRC). However, these studies have been carried out mainly in developed countries where CRC has a high incidence, and it is unclear whether the host-microbiome relationships deduced from these studies can be generalized to the global population. To test if the documented associations between the microbiome and CRC are conserved in a distinct context, we performed metagenomic and metabolomic association studies on fecal samples from 30 CRC patients and 30 healthy controls from two different locations in India, followed by a comparison of CRC data available from other populations. We confirmed the association of *Bacteroides* and other bacterial taxa with CRC that have been previously reported in other studies. However, the association of CRC with Flavonifractor plautii in Indian patients emerged as a novel finding. The plausible role of *F. plautii* appears to be linked with the degradation of beneficial anticarcinogenic flavonoids, which was also found to be significantly correlated with the enzymes and modules involved in flavonoid degradation within Indian CRC samples. Thus, we hypothesize that the degradation of beneficial flavonoids might be playing a role in cancer progression within this Indian cohort. We also identified 20 potential microbial taxonomic markers and 33 potential microbial gene markers that discriminate the Indian CRC from healthy microbiomes with high accuracy based on machine learning approaches.

**IMPORTANCE** This study provides novel insights on the CRC-associated microbiome of a unique cohort in India, reveals the potential role of a new bacterium in CRC, and identifies cohort-specific biomarkers, which can potentially be used in noninvasive diagnosis of CRC. The study gains additional significance, as India is among the countries with a very low incidence of CRC, and the diet and lifestyle in India have been associated with a distinct gut microbiome in healthy Indians compared to other global populations. Thus, in this study, we hypothesize a unique relationship between CRC and the gut microbiome in an Indian population.

## Abstract (precis)

This study provides novel insights on the CRC-associated microbiome of a unique cohort in India, reveals the potential role of a new bacterium in CRC, and identifies cohort-specific biomarkers, which can potentially be used in noninvasive diagnosis of CRC. The study gains additional significance, as India is among the countries with a very low incidence of CRC, and the diet and lifestyle in India have been associated with a distinct gut microbiome in healthy Indians compared to other global populations. Thus, in this study, we hypothesize a unique relationship between CRC and the gut microbiome in an Indian population.

## INTRODUCTION

Colorectal carcinoma (CRC) is among the most frequently diagnosed cancers worldwide and is a major cause for mortality (1). CRC shows the highest incidence in developed countries, such as in the United States and Japan, and is also on the rise in East Asian countries. Mutations in several tumor suppressor genes, such as APC, MSH2, MLH1, PMS2, DPC4/Smad4, and p53, and activation of oncogenes, such as β-catenin, COX-2, and K-RAS, have been implicated as one of the many causes of colorectal cancer (2). The human colon is a unique organ that harbors thousands of bacterial species comprising ∼10^12^ to 10^−14^ microbes, which play a prominent role in human health, likely implicated in the etiology of several human diseases such as inflammatory bowel disease (IBD), obesity, type 2 diabetes, and cardiovascular and other diseases (3–5). Similar associations of an altered gut microbiome with CRC have also been revealed in recent studies in Chinese, Austrian, French, and American populations (6–9). In the majority of the studies, Fusobacterium nucleatum and *Bacteroides* spp. have been observed to be consistently associated with tumorigenesis (7, 10).

Beyond taxonomic profiling, a few recent metagenomic studies have also focused on the identification of potential fecal biomarkers for the improved detection of CRC (6, 8). In the Chinese population, Yu et al. identified 20 microbial gene markers differentiating the CRC and healthy gut microbiome (6). Another study, from a European population, also identified potent taxonomic biomarkers, which showed similar diagnostic accuracy as that of the fecal occult blood test (FOBT) for both early- and late-stage CRC (8). When the two approaches were combined, an improvement of >45% in sensitivity of machine learning models was observed compared to FOBT, while maintaining their specificity for CRC detection, suggesting that microbial biomarkers hold the potential to supplement the existing diagnostic techniques for early-stage and noninvasive detection of CRC.

The previous microbiome studies have mostly emphasized the identification of global CRC markers, as opposed to population-specific microbial biomarkers. However, most of these studies also focus on developed countries and/or populations with high incidences of CRC, which may share environmental or lifestyle factors that influence both CRC and the microbiome. It is, therefore, unclear how universal the reported associations between CRC and the gut microbiome are. Due to the significantly distinct lifestyles and dietary characteristics of different populations worldwide, it is important to identify both country-specific and global markers of CRC.

India is among the few countries in the world where CRC shows the lowest incidence. Low rates of CRC in India are often linked to vegetarianism, use of spices such as curcumin (turmeric), and other food additives having apparent anticancer properties (11). Given the profound role of diet in shaping the gut microbiome, these unique dietary traits are likely to affect the gut microbiome. A cross-population comparison carried out in one of our recent studies also showed that the Indian population forms a distinct cluster from other world populations (China, United States, and Denmark), driven by the predominance of *Prevotella* spp. (12). If the gut microbiome does mediate CRC disease progression, these unique gut microbial traits may explain the low incidence of CRC in India. However, no study has yet been carried out in the Indian population to examine these relationships. Therefore, to gain novel insights into the role of the gut microbiome in CRC in India, and to identify population-specific bacterial markers of CRC, we performed a comprehensive gut microbiome analysis of CRC patients in India and compared that microbiome with healthy Indian individuals. Specifically, we profiled the fecal metagenome using shotgun metagenomic sequencing along with gas chromatography-mass spectrometry (GC-MS)-based profiling of the fecal metabolome in a cohort of 60 individuals (30 CRC patients and 30 healthy controls) from two distinct locations (north-central and southern India).

## RESULTS

Shotgun metagenomic sequencing in *n* = 60 individuals from both Bhopal and Kerala cohorts (see Table S1 in the supplemental material) yielded a total of 641 million high-quality sequencing reads with an average of 10.7 ± 5.1 million reads/sample (average ± SD). We then constructed a gene catalogue containing a set of 2,364,248 nonredundant genes for the Indian cohort. For maximum quantification of microbial genes, the Integrated Gene Catalogue (IGC) and the India-specific gene catalogue were combined to construct a nonredundant Updated Gene Catalogue (UGC), which comprised 11,118,467 genes (an addition of 12.5% genes in the current IGC) including 9,879,896 genes from the IGC and 1,238,571 genes unique to the Indian population. The UGC was used for mapping of metagenomic reads from the 60 Indian samples and resulted in 54.47% ± 7.84% (average ± SD) mapping of reads and in the identification of 3,824,855 million genes in the Indian cohort.

10.1128/mSystems.00438-19.4 TABLE S1 Supplementary data containing the metadata and sample information. Download Table S1, XLSX file, 0.02 MB. Copyright © 2019 Gupta et al. 2019 Gupta et al. This content is distributed under the terms of the Creative Commons Attribution 4.0 International license.

### Variations in the CRC-associated gut microbiome in the Indian cohort.

**FIG 1:**
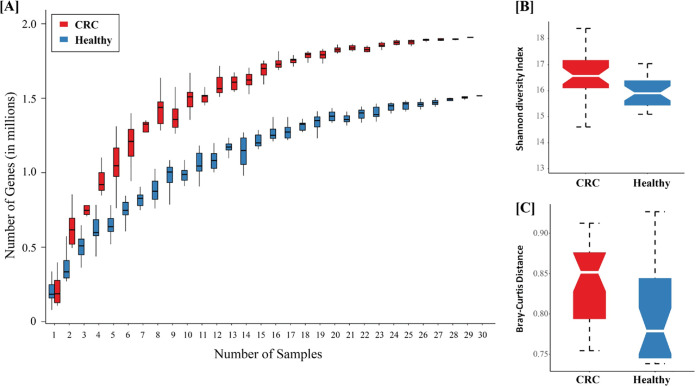
Variations in intersample and intrasample diversity between healthy and CRC samples. (A) Rarefaction curves based on gene counts at each sample depth in healthy and CRC individuals are shown using the box plot. (B) Richness of microbial communities revealed using Shannon diversity is shown for healthy and CRC samples. (C) Intersample Bray-Curtis distances showing diversity between CRC and healthy samples are shown using the box plot. The boxes represent interquartile range between the first (25th percentile) and third (75th percentile) quartiles, and the line or notch in the boxes represents the median. The whiskers extending 1.5× interquartile range on both sides represent the deviations in the values from the median.

Rarefaction analysis showed that the gene richness approached saturation in both groups (healthy and CRC) and was higher in CRC than in healthy individuals (Fig. 1A). The increased gene richness was further validated by calculating the within-sample diversity (α-diversity) using the Shannon index, which measures within-sample gene diversity. It was observed that the individuals with CRC had a more significantly diverse gene pool than healthy controls (Wilcoxon rank sum test; *q* value = 0.0052) (Fig. 1B). Interindividual distances in gene composition, as determined by Bray-Curtis distance metrics, showed that CRC individuals are much more dissimilar than healthy controls (Wilcoxon rank sum test; *q* value = 0.0003) (Fig. 1C). Taken together, these results suggest distinct differences in the diversity of functions carried out by gut microbial communities in the CRC-associated gut compared to the healthy controls.

To compare the gene contents among all 60 samples, a set of genes commonly present in at least 3 samples (5% of the total samples) was constructed, which comprised 1,988,680 genes. Using these 1.9 million genes, gene abundance profiles were generated for each of the 60 samples. The variations in microbial community composition between samples were first scored to examine the effect of each of 8 covariates (health status, location, age, gender, body mass index [BMI], stage, histopathology, and localization) (Table 1) by performing permutational multivariate analysis of variance (PERMANOVA) on the gene abundance profiles. It was observed that health status explained the maximum variation (*P* value = 0.0009, *R*^2^ = 0.04) compared to the other covariates. The location also showed a significant effect but explained less variation than the health status (*P* value = 0.009, *R*^2^ = 0.03).

**TABLE 1:** PERMANOVA on microbial gene profiles of all samples to test the impact of health status, sample location, and clinical parameters on the gut microbiota with *q* of <0.01 (in bold)^*a*^

Variable	df	S.Sq	F.Model	*R*^2^	Pr (>*F*)	*q* value
Health status	1	1.22	2.97	0.04	**0.0009**	**0.004**
Age	1	0.506	1.24	0.02	0.03	0.09
Gender	1	0.408	0.99	0.01	0.467	0.6
BMI	1	0.444	1.08	0.01	0.183	0.336
Location	1	0.896	2.19	0.03	**0.0009**	**0.004**
TNM staging	1	0.443	1.08	0.02	0.1878	0.336
Histopathology	1	0.343	0.84	0.01	0.972	0.972
Localization	1	0.411	1.00	0.01	0.409	0.6

a df, degrees of freedom; S.Sq, sum of squares; F.Model, F-statistic; Pr, *P* value; *R*^2^, coefficient of determination.

To further dig into the covariates explaining variation in the gene profiles across cohorts, principal-component analysis (PCA) based on the gene profiles was performed. The first and the second principal component explained 7.2% and 6.8% of the total variations (see Fig. S1 in the supplemental material) and were significantly associated with health status (polyserial correlation; *q* value < 10^−15^) and location (polyserial correlation; *q* value = 0.00004), respectively (Table S2). CRC and healthy samples clustered separately along PC1, corroborating significant functional microbiome differences explained mainly by the health status followed by location of the samples (Table S2).

10.1128/mSystems.00438-19.1 FIG S1 Principal-component analysis using raw gene abundances of all 60 samples. Download FIG S1, PDF file, 0.2 MB. Copyright © 2019 Gupta et al. 2019 Gupta et al. This content is distributed under the terms of the Creative Commons Attribution 4.0 International license.

10.1128/mSystems.00438-19.5 TABLE S2 Polyserial correlation of the covariates with 10 principal components, calculated using microbial gene profiles from 60 samples using 1.9 million genes. Download Table S2, DOCX file, 0.01 MB. Copyright © 2019 Gupta et al. 2019 Gupta et al. This content is distributed under the terms of the Creative Commons Attribution 4.0 International license.

### Taxonomic variations in the CRC-associated gut microbiome.

**FIG 2:**
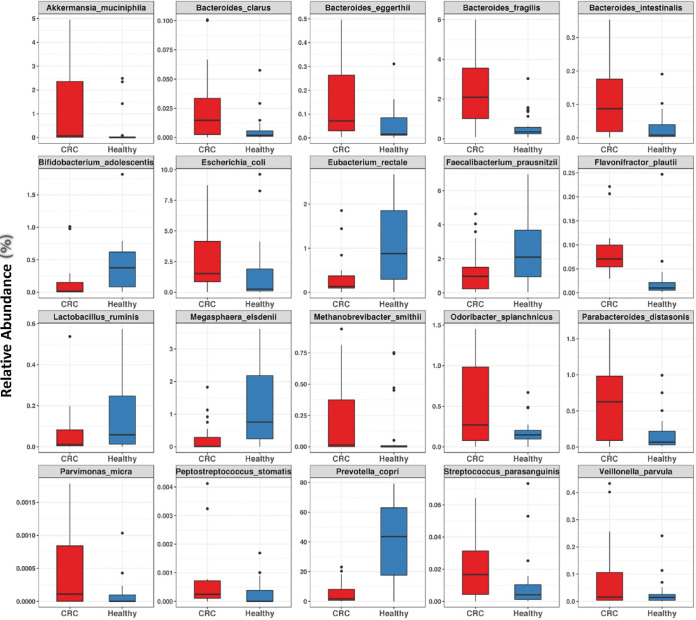
The 20 differentially abundant species between healthy and CRC samples. The box plots showing 20 differentially abundant species observed using all three methods of species identification between CRC (red) and healthy (blue) samples are represented in panels. The *y* axis represents relative abundance of samples calculated by mapping the reads against reference genomes collected from HMP-NCBI. The boxes represent interquartile ranges between the first (25th percentile) and third (75th percentile) quartiles, and the line or notch in the boxes represents the median. The whiskers extending 1.5× interquartile range on both sides represent the deviations in the values from the median.

Taxonomic differences in the gut microbiome of CRC and healthy individuals were examined to identify the microbial taxa associated with the patterns observed in the previous analysis. For this analysis, three different methods were used: (i) reference-based Human Microbiome Project-National Center for Biotechnology Information (HMP-NCBI) species, (ii) *de novo* clustering-based metagenomic species (MGS), and (iii) clade-specific-marker-based metagenomic OTU (mOTU) species and Metaphlan species (see Materials and Methods). On performing correlation analysis, 158 HMP-NCBI-mapped species, 147 MGS, 61 species-level mOTUs, and 45 Metaphlan species were observed to be significantly associated with CRC or healthy samples (Wilcoxon rank sum test; *q* value < 0.01; mean abundance > 0.001) (Table S3). To improve the robustness of taxonomic marker identification in CRC, the taxonomic species that were identified by all the three strategies simultaneously (HMP-NCBI species, MGS, and any one of the clade marker-based approaches, i.e., mOTUs or Metaphlan) were considered for further analysis. A total of 20 taxonomic markers were identified based on their significant association with the health status using the above methods. Among these 20 marker species, six species, namely, Eubacterium rectale, Prevotella copri, Bifidobacterium adolescentis, Megasphaera elsdenii, Faecalibacterium prausnitzii, and Lactobacillus ruminis, were observed to be highly associated with the gut microbiome of healthy Indian subjects. These species have also been associated with a healthy phenotype in previous studies, and significant reductions in their proportion were observed in CRC in this study (13–18). The remaining 14 species were associated and enriched in CRC samples. Among these, nine species including Akkermansia muciniphila (6, 19), Bacteroides fragilis (20), Bacteroides clarus (21), Bacteroides eggerthii (7), Escherichia coli (6, 19), Odoribacter splanchnicus (7), Peptostreptococcus stomatis (6, 8), Parvimonas micra (6, 7), and Parabacteroides distasonis (22), have been shown to be strongly associated with colorectal cancer in the previous studies (Fig. 2).

10.1128/mSystems.00438-19.6 TABLE S3 Correlation analysis of HMP-NCBI mapped species, MGS, species-level mOTUs, and Metaphlan species with health status. Download Table S3, XLSX file, 0.2 MB. Copyright © 2019 Gupta et al. 2019 Gupta et al. This content is distributed under the terms of the Creative Commons Attribution 4.0 International license.

Remarkably, a few gut bacteria that have not yet been associated with colorectal cancer in the previous reports were also observed to be significantly associated with Indian CRC samples. Among these, a novel and striking finding was the presence of *Flavinofractor plautii*, which was significantly associated (Wilcoxon rank sum test, *q* < 0.00001) (Fig. 2) with CRC samples in this study. Additionally, the predictive power of taxonomic association using Random Forest (RF) analysis on HMP-NCBI species abundance also showed Flavonifractor plautii as the most important species in separating Indian CRC samples from the healthy controls (Table 2). The high abundance of this flavonoid-degrading bacterium in Indian samples is intriguing, as the diet of Indian populations is rich in polyphenols, with flavonoids being the most abundant dietary polyphenol (23). Additionally, a few gut bacteria not associated with colorectal cancer in the previous reports were also observed to be highly associated with CRC; these included Bacteroides intestinalis, Methanobrevibacter smithii, Streptococcus parasanguinis, and Veillonella parvula (Fig. 2).

**TABLE 2:** Mean decrease in accuracy calculated using the 20 marker species

Species	Mean decrease	
	Accuracy	Gini
Flavonifractor plautii	0.0688	5.0677
Prevotella copri	0.0313	2.5754
Peptostreptococcus stomatis	0.0144	1.2526
Bacteroides intestinalis	0.0128	1.3198
Bifidobacterium adolescentis	0.0104	1.0669
Bacteroides fragilis	0.0097	1.4901
Streptococcus parasanguinis	0.0085	0.9585
Odoribacter splanchnicus	0.0067	0.9543
Parvimonas micra	0.0061	0.7280
Eubacterium rectale	0.0061	0.7019
Bacteroides eggerthii	0.0054	0.6979
Escherichia coli	0.0047	0.8368
Veillonella parvula	0.0043	0.6180
Akkermansia muciniphila	0.0039	0.5529
Methanobrevibacter smithii	0.0033	0.4000
Parabacteroides distasonis	0.0031	0.4248
Megasphaera elsdenii	0.0029	0.7362
Lactobacillus ruminis	0.0028	0.5452
Faecalibacterium prausnitzii	0.0024	0.3228
Bacteroides clarus	0.0020	0.3583

**FIG 3:**
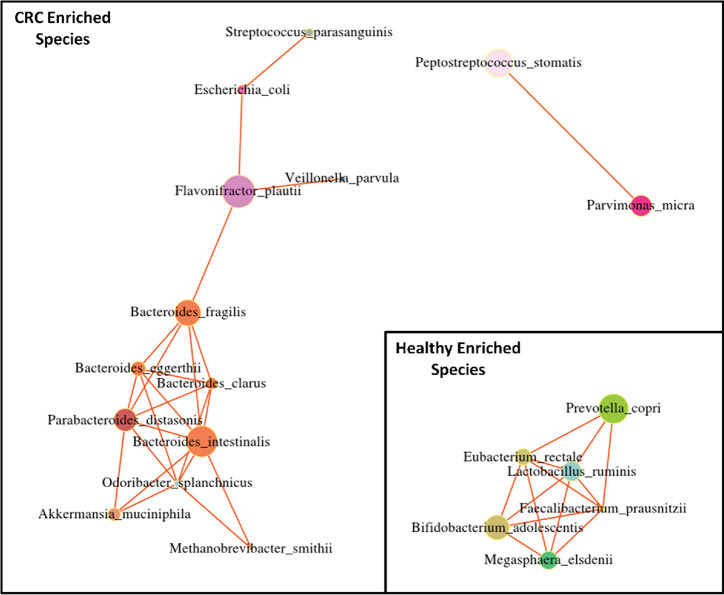
Species involved in gut microbial dysbiosis associated with colorectal cancer. The cooccurrence network derived from Spearman’s rank correlation coefficient using the relative abundance of 20 differentially abundant species is shown. The 14 species which are enriched in CRC individuals and 6 species which are enriched in healthy individuals are shown. Significant correlations (ρ > 0.5 and FDR-adjusted *P* < 0.05) are shown using network analysis. The node size shows the association of each species with other species. The node color shows the taxonomic families to which they belong.

Further, PERMANOVA showed that only health status (*q* value = 0.004) and location (*q* value = 0.004) significantly explained variation in species abundance based on the four methods used to identify species. All other covariates were not significant (*q* value > 0.09) (Table S4). In order to derive associations between microbial markers, a species cooccurrence network was generated from pairwise correlations using sparCC (Fig. 3). Flavonifractor plautii, Bacteroides fragilis, Bacteroides intestinalis, and Parabacteroides distasonis, which have been previously reported to be associated with CRC, showed higher degrees of association with each other and also with microbes such as Peptostreptococcus stomatis and Parvimonas micra (6). The most influential nodes in the network were determined using centrality measure, and it was observed that Flavonifractor plautii, Bacteroides intestinalis, Bacteroides fragilis, Bacteroides clarus, and Parabacteroides distasonis showed much higher centrality, thus showing their influence on the entire network. The high association between these species indicates that CRC-associated microbes tend to cooccur, and form more associations, in contrast with taxa characterizing healthy states.

10.1128/mSystems.00438-19.7 TABLE S4 PERMANOVA of taxonomic species identified using four different strategies to test the impact of health status, sample location, and clinical parameters on the gut microbiota with *q* of <0.01. Download Table S4, DOCX file, 0.01 MB. Copyright © 2019 Gupta et al. 2019 Gupta et al. This content is distributed under the terms of the Creative Commons Attribution 4.0 International license.

### Global comparative metagenome-wide association study (MGWAS) meta-analysis.

**FIG 4:**
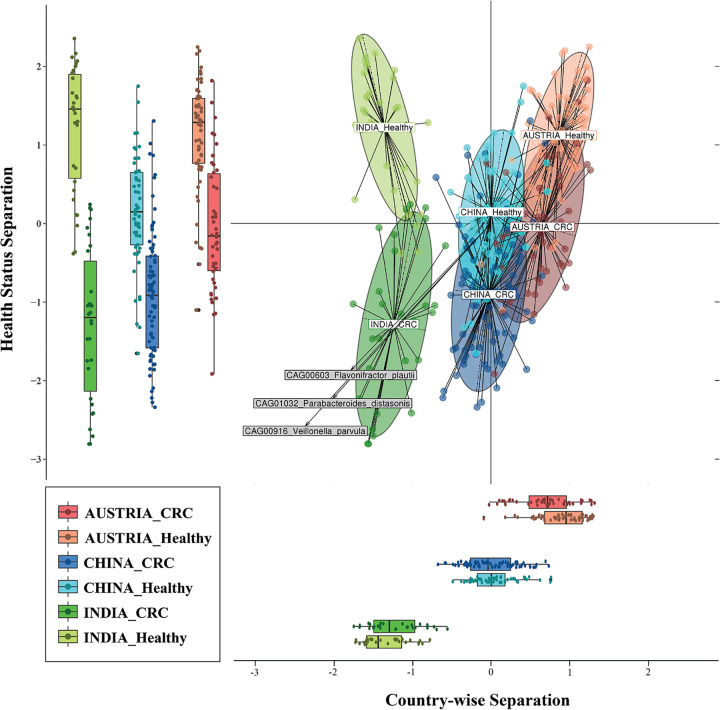
Major effects of CRC on gut microbiome from multivariate meta-analysis. Principal-component analysis of the samples from China, Austria, and India using MGS abundance derived from metagenome-wide association study is projected. The multivariate analysis using distance-based redundancy analysis (db-RDA) was constrained by studies/populations and health status. The marginal box plots show separation of constrained projected coordinates on the *x* axis (constrained for studies/populations) and *y* axis (constrained for health status). The top three MGS that showed significant association with Indian CRC samples are interpolated on the plane of maximal separation.

To demonstrate the utility of CRC-associated taxonomic markers in the CRC-associated gut microbiome between cohorts, we selected a similar group of 75 CRC cases and 53 healthy control samples from a Chinese cohort (19), and a group of 46 CRC cases and 57 healthy control samples from an Austrian cohort (7) for the comparative analysis. Using data sets from multiple countries, a meta-analysis was performed to identify global variations in the CRC microbiome. In order to control the variations arising due to uneven sequencing depths from other studies, we used a rarefied table for performing meta-analysis. We performed multivariate distance-based redundancy analysis (db-RDA) with health status (CRC and healthy) and country (India, China, and Austria) as metainformation. The multivariate analysis was constrained using these two pieces of information, and the most important axes explaining maximum variations between samples were extracted. The projection shows all the CRC and healthy samples from the three countries, with country-/study-wide differences on the *x* axis and differences due to CRC status on the *y* axis (Fig. 4). It was observed that the Indian population differed significantly from the Austrian and Chinese cohorts (*P* value < 10^−15^) (Fig. 4). The Indian CRC samples showed marked differences in microbial composition and were separated from the other country samples, thus revealing the unique microbial community composition in Indian gut microbiomes. The MGS/CAGs that showed maximum contributions in driving the separation of Indian CRC samples included Flavonifractor plautii, Veillonella parvula, and Parabacteroides distasonis (Fig. 4).

To look at the global taxonomic patterns, we performed differential analysis unstratified for CRC status (while controlling for the populations) and found 85 MGS/CAGs to be significantly associated with CRC (Table S5). The MGS/CAG belonging to Fusobacterium nucleatum, which has been reported in earlier studies (24), showed the highest association with CRC status with a *P* value of <10^−15^. The other species that have been associated with CRC in the previous studies included Peptostreptococcus stomatis, Bacteroides fragilis, and Porphyromonas asaccharolytica (6, 7, 25). Flavonifractor plautii, which showed a striking association in Indian CRC samples, was also observed in this list, albeit with low *P* values compared to the previously mentioned species.

10.1128/mSystems.00438-19.8 TABLE S5 Differential analysis using MGS/CAGs unstratified for CRC status. Download Table S5, XLSX file, 0.01 MB. Copyright © 2019 Gupta et al. 2019 Gupta et al. This content is distributed under the terms of the Creative Commons Attribution 4.0 International license.

### Functional characterization of microbiome associated with CRC.

A metagenome-wide association analysis was performed to gain functional insights on the CRC-associated gut microbiome. Out of the total of 1.9 million genes, which were present in at least 5% of the samples, 228,299 genes were found to be significantly associated with the disease status (Wilcoxon rank sum test, *q* < 0.01). These CRC-associated genes were functionally annotated using the KEGG database. Using the stringent criteria of *P* value of <0.01 and log odds ratio (LOR) of >|2|, 473 KEGG orthologues (KOs) were found associated with health status (Table S6). The top-ranked enzymes (KOs) include invasins, multidrug resistance protein, and enzymes involved in secretion and the transport system, which points toward a pathogenic and invasive environment with high cross-talk between host and microbes. Specifically, the high abundance of invasins has also been associated with the colorectal cancer-associated gut microbiome in the past (26, 27), as they help the bacteria to gain entry into host cells (22, 26).

10.1128/mSystems.00438-19.9 TABLE S6 List of 473 KOs found to be significantly associated with CRC status. Download Table S6, XLSX file, 0.04 MB. Copyright © 2019 Gupta et al. 2019 Gupta et al. This content is distributed under the terms of the Creative Commons Attribution 4.0 International license.

The pathways associated with CRC were identified using reporter feature analysis, which takes into consideration the significance and enrichment of all the genes present in the pathway. It was observed that out of the 12 pathways, “ABC transporters” (*q* value = 0.013) could pass the stringent cutoff being significantly enriched in CRC (Table S7). It was interesting that pathways related to the biosynthesis of six amino acids (leucine, isoleucine, lysine, phenylalanine, tryptophan, and valine) out of the nine essential amino acids were observed to be significantly high in healthy controls compared to the CRC cases (Table S7), suggesting depletion of essential amino acids in the gut microbiome of CRC individuals.

10.1128/mSystems.00438-19.10 TABLE S7 List of pathways significantly associated with heath status identified using reporter feature analysis. Pathways in red are abundant in CRC individuals, and pathways in green are abundant in healthy individuals. Download Table S7, XLSX file, 0.01 MB. Copyright © 2019 Gupta et al. 2019 Gupta et al. This content is distributed under the terms of the Creative Commons Attribution 4.0 International license.

Further, to gain additional functional insights, we identified KEGG modules which were significantly associated with health status. For this, only those modules for which at least 90% of the module’s enzymes are present in the samples were considered, and these modules were also found associated with health status with a *q* value of <0.001 (Wilcoxon rank sum test). Using these stringent criteria, a total of 46 modules could qualify, of which 12 modules were found higher in CRC cases than in the healthy controls. A module with the function of “Catechol ortho cleavage” was observed to be significantly associated with the CRC cases. This module is involved in the degradation of catechols such as 3,4-dihydroxypheynlacetic acid, which are generated by degradation of flavonoids by the gut bacterium Flavonifractor plautii (28). On performing the Spearman correlation between these 46 modules and the 20 taxonomic species selected above, the “Catechol ortho cleavage” module was observed to correlate significantly (*r* = 0.63, *P* = 3.6 × 10^−7^) with Flavonifractor plautii (Fig. S2).

10.1128/mSystems.00438-19.2 FIG S2 Association of differentially abundant modules in CRC and healthy samples with the 20 taxonomic markers associated with health status. Download FIG S2, PDF file, 0.2 MB. Copyright © 2019 Gupta et al. 2019 Gupta et al. This content is distributed under the terms of the Creative Commons Attribution 4.0 International license.

### Insights from comparative metabolomic profiling.

**FIG 5:**
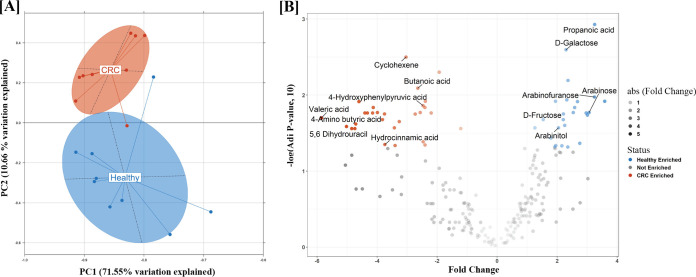
(A) Principal-component analysis of the raw metabolomic peaks identified from CRC and healthy samples (*n* = 18). The PC1 and PC2 explaining almost 80% of variation between samples show distinct metabolomic profiles in CRC and healthy individuals. (B) Volcano plot showing the significantly enriched metabolites in CRC and healthy individuals. The fold change was calculated as log fold between CRC and healthy individuals. The adjusted *P* values are plotted on the *y* axis. The metabolites significantly enriched in CRC patients (adjusted *P* value < 0.05; fold change < −2) are shown in red whereas those enriched in healthy individuals (adjusted *P* value < 0.05; fold change > 2) are shown in blue. Valeric acid, butanoic acid, and 4-hydroxyphenyl acetic acid were observed to be significantly higher in CRC patients than in healthy individuals.

The principal-component analysis revealed marked variations in the metabolomic profiles of CRC and healthy individuals. These differences could be attributable to the host physiological changes and the microbial metabolism, which depends on the type of microbes inhabiting the gut. The first and the second principal components explained 71.55% and 10.66% of the total variations (Fig. 5A), respectively. The peaks annotated to metabolites 4-hydroxyphenyl pyruvic acid, butanoic acid, valeric acid, l-valine, and cyclohexene were observed to be significantly enriched (*P* value < 0.05; log fold change < −2) in CRC individuals compared to the healthy group (Fig. 5B). Among these, the higher level of 4-hydroxyphenyl derivates can be directly corrected with the flavonoid-metabolizing ability of the CRC microbiome, which is dominated by Flavonifractor plautii (formerly Clostridium orbiscindens), a microbe involved in the degradation of quercetin flavonoid (28, 29). A higher abundance of compounds such as valerate, isovalerate, and isobutyrate (which are salts or esters of valeric acid and butanoic acid [butyric acid]) in CRC individuals has also been reported in other studies (30). Taken together, these observations indicate significant differences in the metabolic profiles in CRC individuals compared to the healthy group, which correlates with the flavonoid-metabolizing ability of the CRC microbiome in the Indian patient cohort. However, detailed studies to gain insights on the functional contribution of respective microbes for production of these metabolites and their impact on host health are needed to confirm these results.

### CRC gene biomarker discovery.

**FIG 6:**
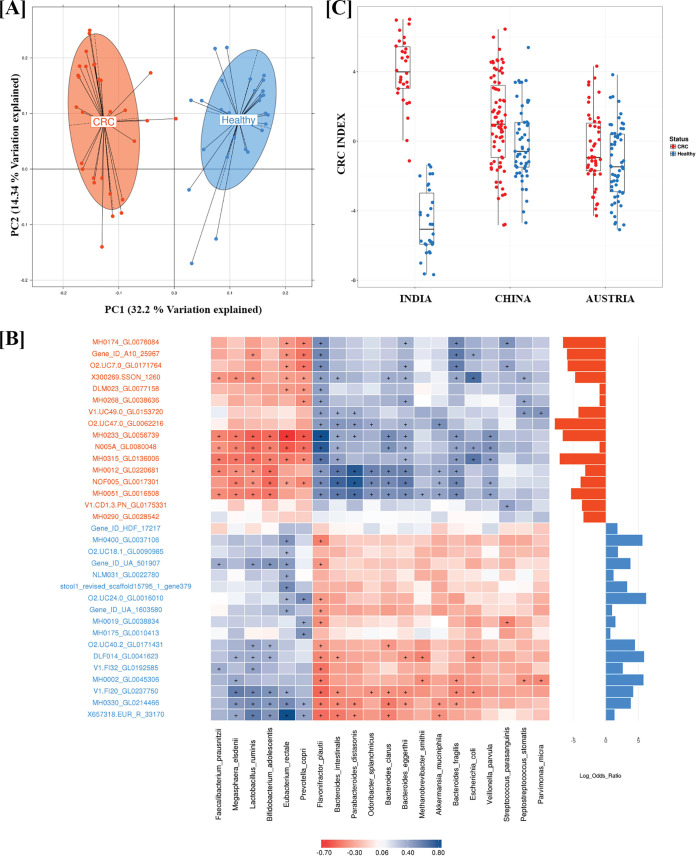
Association of 33 gene markers with the health status and taxonomic species identified. (A) Principal-component analysis based on abundances of 33 gene markers explains 40% of variation using only the first two principal components. (B) Association of 33 gene markers with the 20 taxonomic species identified using three different strategies. (C) CRC index computed using the log abundances of the 33 gene markers showing significant association only in the Indian CRC and healthy samples compared to Chinese and Austrian cohorts.

We divided our 60 samples into two sets, cohort A comprising 48 samples and cohort B comprising 12 samples, by random selection from the two locations and health status (see Materials and Methods). To identify potential CRC-associated biomarkers, a robust feature selection method was followed using the 102,168 health status-associated genes from the samples of cohort A. From these genes, we identified a subset that were highly correlated with each other (Pearson correlations ρ > 0.9) and chose the longest gene from each correlated group to construct a statistically nonredundant set of 13,982 genes. Further, we used the “CfsSubsetEval” method from Weka to identify a subset of 36 genes that are highly correlated with the health status while having low intercorrelation with each other. The genes from this subset were further validated using the Boruta algorithm, which uses Random Forest to perform a top-down search for relevant features by comparing original attribute importance with importance achievable at random, and we eliminated irrelevant feature to stabilize the test. As a result, 33 out of 36 genes were confirmed as markers using this algorithm and 3 were predicted as tentative markers (Fig. S3). The principal-component analysis using these 33 genes showed clear separation between CRC and healthy samples, and the first two principal components explained 40.5% variation (Fig. 6A), which is a significant improvement compared to the separation observed using raw data (11.4% variation explained using first two principal components [Fig. S1]). Most importantly, the first three principal components were observed to be significantly (adjusted *P* values: PC1 = 7.5 × 10^−10^, PC2 = 1.97 × 10^−8^, PC3 = 0.0005) associated only with the health status with the stringent *P* value cutoff less than 0.001 (Table 3). PERMANOVA showed that only CRC status explained the variation in the 33 marker gene abundances significantly (*P* < 0.01, *R*^2^ = 0.19) (Table 4). These results suggest that the 33 gene markers identified using the approach are strongly associated with health status and not with any other covariate. Further, to evaluate the predictive power of these marker genes in predicting the CRC status, the Random Forest method was used, which resulted in the perfect classification of the two classes (area under receiver operating characteristic [ROC] curve, area under the curve [AUC] = 1) using 10-fold cross-validation. On performing the Spearman correlation of these 33 gene markers with the 20 taxonomic markers identified above, it was observed that the 16 genes enriched in CRC cases were highly correlated with Flavonifractor plautii and Bacteroides fragilis (Fig. 6B). These results further validates that these two species could play a role in Indian CRC samples.

**TABLE 3:** Polyserial correlation of first five principal components with the 33 gene markers identified in this study

Principal component and covariate	Z score	*P* value	Adjusted *P* value (Benjamini-Hochberg)^*a*^
PC1			
Status	−6.4038	1.52E−10	**7.5 × 10^−10^**
Age	2.3870	0.01699	0.0424
Gender	1.2505	0.21113	0.263
BMI	−1.4470	0.14789	0.2464
Location	0.7238	0.46920	0.469
PC2			
Status	5.7314	9.96E−09	**4.97 × 10^−8^**
Age	−1.6525	0.09843	0.099
Gender	−2.1843	0.02894	0.0482
BMI	1.6496	0.09902	0.099
Location	2.5625	0.01039	0.0259
PC3			
Status	−3.8852	0.00010	**0.0005**
Age	2.1147	0.03445	0.057
Gender	0.7851	0.43240	0.432
BMI	−1.4414	0.14948	0.1868
Location	2.8859	0.00390	0.0097
PC4			
Status	0.0925	0.92628	0.9262
Age	0.4996	0.61738	0.7717
Gender	0.9185	0.35837	0.608
BMI	−0.9062	0.36485	0.608
Location	−1.7567	0.07898	0.394
PC5			
Status	−1.8325	0.06688	0.3343
Age	−0.2352	0.81408	0.81407
Gender	0.5581	0.57681	0.721
BMI	−1.0634	0.28759	0.7189
Location	−0.7609	0.44673	0.721

a The boldface values represent significant associations defined as *P* value < 0.001.

**TABLE 4:** PERMANOVA of 33 gene markers to test whether these 33 markers are significantly associated with health status and not with any other covariate^*a*^

Parameter	df	Gene marker				
		S.Sq	F.Model	*R*^2^	Pr (>*F*)	*q* value
Health status	1	3.9072	15.4497	0.1866	0.0099	**0.0495**
Age	1	0.4392	1.7369	0.0209	0.0297	0.8910
Gender	1	0.3200	1.2654	0.0152	0.1584	0.3168
BMI	1	0.2980	1.1784	0.0142	0.2574	0.3168
Location	1	0.6581	2.6022	0.0314	0.0198	0.0792

a df, degrees of freedom; S.Sq, sum of squares; F.Model, F-statistic; Pr, *P* value; *R*^2^, coefficient of determination.

10.1128/mSystems.00438-19.3 FIG S3 Results from the Boruta analysis showing 33 genes out of 36 as confirmed markers and 3 genes as intermediate markers. Blue box plots correspond to minimal, average, and maximum Z score of a shadow taxon. Yellow and green boxplots represent Z scores of tentative and confirmed taxa, respectively. Download FIG S3, PDF file, 0.4 MB. Copyright © 2019 Gupta et al. 2019 Gupta et al. This content is distributed under the terms of the Creative Commons Attribution 4.0 International license.

### Gene marker validation in independent metagenomic cohorts.

To test the accuracy and robustness of these gene markers, we evaluated the predictive power of these 33 genes on cohort B (6 CRC samples and 6 healthy samples) from this study and from a cohort with a different genetic background: 75 CRC samples and 53 healthy samples from China and 46 CRC samples and 57 healthy samples from Austria. The relative gene abundances of China and Austria data sets were constructed by mapping their genes on the Updated Gene Catalogue constructed in this study. On cohort B, the Random Forest (RF) model using the 33 genes resulted in an accuracy of 91.67% with 11 out of 12 samples being correctly predicted (sensitivity, 100%; specificity, 83.33%). However, using the same gene markers on the Austria and China data sets resulted in lower average accuracy of 65.05% and 51.56%, respectively. A CRC index using the log relative abundances of the 33 gene markers was also calculated as mentioned in the study by Yu et al. (6). The CRC index clearly separated the samples from the Indian population (CRC index patients = 4.04; CRC index healthy = −4.65) with a *P* value of 3 × 10^−1.^ However, it could not significantly differentiate between the CRC cases and healthy controls for the other two populations (Fig. 6C).

## DISCUSSION

Recently, gut microbiome dysbiosis has emerged as a key factor that triggers an inflammatory response in the host and is proposed to lead to an initiation of colorectal cancer (31, 32). However, most of our understanding comes from developed countries with high incidences of CRC. India harbors a unique gut microbiome and also has one of the lowest incidences of CRC. Thus, we expected to find a distinct relationship between the gut microbiome and CRC in an Indian cohort. Our results showed a clear distinction between the healthy and CRC-associated gut microbiomes. We also identified multiple potential microbial taxonomic and gene biomarkers associated with CRC. While some of these biomarkers have been reported in other global populations, others were unique to our cohort. Therefore, our study is one of the first to emphasize the importance of utilizing population-specific microbiome biomarkers in studies of CRC.

Interestingly, gut microbiome diversity was higher in Indian CRC samples than in healthy controls. A similar observation was made in Austrian CRC cases, which showed an increased microbiome diversity; however, reduced microbial diversity was observed in a Chinese cohort, pointing toward a contrasting trend, perhaps due to population-specific variations (12). The higher diversity in the Indian CRC microbiome can be explained by the fact that the Indian gut microbiome is highly skewed, with most (30% to 75%) of the community dominated by *Prevotella*, as observed in our recent study that examined the gut microbiome in a cohort of 110 Indian individuals (12). In this study, we observed a much lower proportion of *Prevotella* in CRC samples (12.7%) than in the healthy samples (45.31%). Thus, the apparent ∼3.5-fold reduction in this most predominant taxon in CRC patients is likely to result in an increased diversity due to more opportunities for other bacterial taxa to flourish (33). Another consequence of this dysbiosis appears to be the reduction in pathways related to the biosynthesis of six essential amino acids (leucine, isoleucine, lysine, phenylalanine, tryptophan, and valine) out of the nine essential amino acids in CRC cases (see Table S6 in the supplemental material), which makes it tempting to speculate on a dysbiosis-mediated mechanism of CRC in the Indian population.

The most interesting key finding of this study was the identification of Flavonifractor plautii as the key bacterium associated with CRC, which also emerged as one of the 20 taxonomic markers identified using three different strategies. Though its presence in the gut microbiome is not unique to India, and it was present in other CRC data sets (used in this study), it showed a differential abundance only in the CRC gut microbiome of the Indian cohort. In addition to being significantly abundant, it also emerged as the most important species in separating CRC samples from healthy samples in the Indian cohort. Also, the high correlation of *F. plautii* with the 16 CRC-associated gene markers highlights it as a potential key species in the CRC-associated Indian gut microbiome. Among other species that showed a strong association with CRC, the Bacteroides intestinalis and Methanobrevibacter smithii species were observed to be associated with Indian CRC cases and were not previously reported in other CRC microbiome studies*. B. intestinalis* is a gut commensal bacterium known to convert primary bile acids to secondary bile acids via deconjugation and dehydration (34). These secondary bile acids may have carcinogenic effects (35). *M. smithii* is a methanogenic archaeon and a dominant methanogen in the distal colon of both healthy and diseased individuals (36). To date, no direct mechanistic link has been established between gut-associated diseases and methanogens; however, colonization by archaea has been suggested to promote a number of gastrointestinal and metabolic diseases such as colorectal cancer, inflammatory bowel disease, and obesity (37).

*F. plautii* can degrade flavonoids by cleaving the C-ring of the flavonoid molecules (28). Flavonoids are important constituents of the human diet and are mainly comprised of polyphenolic secondary metabolites with broad-spectrum pharmacological activities. Accumulating evidence from epidemiological, preclinical, and clinical studies supports a role of these polyphenols in the prevention of cancer, cardiovascular disease, type 2 diabetes, and cognitive dysfunction (28, 38). Flavonoids are proposed to affect the composition of the gut microbiota and could therapeutically target the intestinal microbiome by promoting beneficial bacteria and inhibiting potentially pathogenic species (28). Several common Indian foods such as tea, coffee, apple, guava, *Terminalia* bark, fenugreek seeds, mustard seeds, cinnamon, red chili powder, cloves, turmeric, and pulses contain large amounts of flavonoids (39). Medium levels (50 to 100 mg) are found in Indian gooseberry, omum, cumin, cardamom, betel leaf, and brandy (39). Small but significant amounts are also present in food items of high consumption such as kidney beans, soybeans, grapes, ginger, coriander powder, millets, and brinjal (39). Given the significance of flavonoids, the high consumption of beneficial flavonoids in the Indian diet has been correlated with low rates of CRC occurrence in India (38).

However, extensive degradation of flavonoids by gut microflora may result in lower overall bioavailability of intact flavonoids (40). Thus, in the Indian CRC samples it is reasonable to associate the high abundance of Flavonifractor plautii, a key flavonoid-degrading bacterium, with higher rates of flavonoid degradation that minimizes the potential beneficial effects and bioavailability of flavonoids in CRC. Further, the high association of *F. plautii* with the catechol cleavage pathway (catechols are generated by degradation of flavonoids) also indicates a potential role of this species in flavonoid degradation in the gut. In addition, the enzyme enoate reductase, which performs the first step of flavonoid degradation, was also found to be significantly abundant in CRC cases compared to healthy samples (Wilcoxon rank sum test; *P* value = 0.045). Taken together, these observations underscore a potential role of this bacterium in degradation of flavonoids in CRC cases.

Interestingly, Fusobacterium nucleatum has been associated with the CRC microbiome in the past in the major studies from other populations. The meta-analysis performed in this study also found F. nucleatum as the top bacterium in the global CRC-associated microbiome studies. However, this bacterium was not present in the list of the 20 taxonomic markers identified in this study. Although its abundance was significantly higher in CRC cases than in healthy controls, the proportional abundance was below the minimum abundance criterion (>0.1%) selected in this study, and hence, it was not included in the list of taxonomic markers. Further, its presence was almost negligible (0.05%) in the Indian samples in comparison to its basal levels in Austrian and Chinese CRC data sets. Hence, it could not appear as a taxonomic marker for Indian CRC samples.

The results of the study also have translational applications in CRC diagnosis. Survival rates in CRC are reported to increase if the cancer is diagnosed and treated at an early stage (41). The standard colonoscopy method used to diagnose CRC is invasive and also expensive, due to which many high-risk individuals are not screened at their initial stages of cancer. The available noninvasive tests, such as the fecal occult blood test, fecal immunochemical test, and DNA-based Cologuard test (42), lack sensitivity and detection of early-stage disease, may provide false-positive results, and also need confirmation due to nonspecific diagnosis (43). Similarly, the molecular subtyping method which is commonly used in cancer research, where cancer subclasses are based on clinically relevant gene expression patterns (44), does not show clear results in CRC (45, 46). Thus, the apparent limitations in the diagnosis of CRC prompt the need for the development of alternative diagnosis methods such as the microbial biomarkers identified in this study and other similar previous studies. The 33 potential gene markers associated with the Indian microbiome samples and their high accuracy (91.67%) in classifying Indian CRC samples from the healthy samples provide a proof of concept for the development of an affordable diagnostic test using fecal microbial gene markers. However, due to the lack of a significant number of samples to represent each of the four stages of CRC, a correlation analysis of the 33 gene markers with the stage of the cancer could not be performed in this study, which would be helpful to identify the early-stage CRC markers. In addition, the robustness of these candidate markers should be further validated on other Indian cohorts with larger numbers of samples and on similar cohorts in other populations, which is presently a limitation of this study and provides impetus for further studies.

## MATERIALS AND METHODS

### Cohort design and subject enrollment.

A considerable sample size consisting of 60 samples (30 cases and 30 controls) was recruited from two different locations (Bhopal and Kerala) in India. For constructing a balanced data set, 15 cases and 15 controls were selected from both the locations. The two selected locations represent different geographies (2,000 km apart) and lifestyles in order to remove the confounding effect of diet and making the observations generalizable for the Indian cohort. Bhopal is a city located in central India and is populated with people from all over the country; hence, samples from here can act as a proxy to represent the diversity of the country. Samples from Kerala were specifically chosen because, among all the other states of India, Kerala has the highest rate of colorectal cancer incidence. The fecal samples were collected only from CRC cases, and those from healthy subjects were taken from a previous study (12). Each fecal sample was collected and immediately transported to the lab at 4°C for further processing. Diagnosis of all the cases was carried out by experienced oncologists at the hospitals through biopsy and colonoscopy. The study exclusion criteria for patients were any previously diagnosed serious medical conditions and recent use of antibiotics, to avoid the effect of confounding factors. Patients with incomplete medical information were also removed from the selected set. Fecal samples were collected prior to colonoscopy in sterile containers.

### Fecal metagenomic DNA extraction.

Metagenomic DNA was isolated from all the fecal samples using the QIAamp stool minikit (Qiagen, CA, USA) according to the manufacturer’s instructions. DNA concentration was estimated by the Qubit HS double-stranded DNA (dsDNA) assay kit (Invitrogen, CA, USA), and quality was estimated by agarose gel electrophoresis. All the DNA samples were stored at −80°C until sequencing.

### Shotgun metagenome sequencing.

The extracted metagenomic DNA was used to prepare the sequencing libraries using the Illumina Nextera XT sample preparation kit (Illumina Inc., USA) by following the manufacturer’s protocol. The sizes of all the libraries were assessed on the Agilent 2100 Bioanalyzer using the Agilent high-sensitivity DNA kit (Agilent Technologies, Santa Clara, CA, USA) and were quantified on a Qubit 2.0 fluorometer using the Qubit HS dsDNA kit (Life Technologies, USA) and by quantitative PCR (qPCR) using Kapa SYBR Fast qPCR master mix and Illumina standards and primer premix (Kapa Biosystems, MA, USA) according to the Illumina suggested protocol. The shotgun metagenomic libraries were loaded on an Illumina NextSeq 500 platform using the NextSeq 500/550 v2 sequencing reagent kit (Illumina Inc., USA), and 150-bp paired-end sequencing was performed at the Next-Generation Sequencing (NGS) Facility, IISER, Bhopal, India.

### Preprocessing of the metagenomic reads.

A total of 150 Gbp of metagenomic sequence data (mean = 1.36 Gb) was generated from 60 fecal samples. The metagenomic reads were filtered using the NGSQC (v2.3.3) toolkit with a cutoff of *q* of ≥20 (47). The high-quality reads were further filtered to remove the host-origin reads (human contamination) from bacterial metagenomic reads, which resulted in the removal of an average of 1% of reads. The reads from each sample were assembled into contigs at a k-mer size of 63 bp using SOAPdenovo (v2.0) (48). The singletons resulting from each sample were pooled, and *de novo* assembly was repeated on the combined set of singleton reads from all samples. The open reading frames (ORFs) from each contig (length of ≥300 bp) were predicted using MetaGeneMark (v3.38) (49). Pairwise alignment of genes was performed using BLAT (v2.7.6), and the genes which had an identity of ≥95% and alignment coverage of ≥90% were clustered into a single set of nonredundant genes, from which the longest gene was selected as the representative ORF to construct the nonredundant gene catalogue.

The Integrated Gene Catalogue (IGC), which represents 1,297 human gut metagenomic samples comprising HMP, MetaHIT and Chinese data sets, was retrieved (50). The gene catalogue constructed from Indian samples was combined with the IGC to construct a nonredundant gene catalogue (using ≥95% identity and ≥90% alignment coverage) and is referred to as “updated IGC” in the subsequent analysis.

### Quantification of gene content.

The quantification of gene content was carried out using the strategy performed by Qin et al. (51) in which the high-quality reads were aligned against the updated IGC using SOAP2 (v2.21) in the SOAP aligner with an identity cutoff of ≥90% (52). Two types of alignments were considered for sequence-based profiling: (i) the entire paired-end read mapped to the gene and (ii) one end of the paired-end read mapped to a gene and other end remained unmapped. In both cases, the mapped read was counted as one copy. Further, the read count was normalized based on length of the gene as *b_i_* = *x_i_*/*L_i_*.

The relative abundance of a gene within the sample was calculated as follows:

$$a_{i}=\frac{b_{i}}{\sum^{\;}j\mathrm{bj}}=\frac{\frac{x_{i}}{L_{i}}}{\sum^{\;}j\frac{\mathrm{xj}}{\mathrm{Lj}}}$$

where *a_i_* is relative abundance of gene in sample *S*, *x_i_* is number of times that gene *i* was detected in sample *S* (the number of mapped reads), *L_i_* is length of gene *i*, *j* is all the genes, and *b_i_* is copy number of gene *i* in sequenced data from sample *S*.

### Construction of updated gene catalogue for gut profiling.

To construct the gene catalogue for gut microbiome profiling, the high-quality sequencing reads were subjected to a de Bruijn graph-based assembly which resulted in 2,143,541 contigs of >300 bp in length with a total contig length of 1.52 Gb. To capture low-coverage genomic regions or low-abundance genomes, all unassembled reads were extracted and combined with the singletons from each sample to further assemble into an additional 1.2 million contigs (>300 bp) with a total assembled length of 0.76 Gb. The gene prediction on all assembled contigs resulted in 4,591,809 genes, out of which 2,36,4248 genes were nonredundant and which represents the gene catalogue of the Indian population. We incorporated these genes to update the currently available Integrated Gene Catalogue (IGC), which contained 9.8 million genes from 1,267 gut metagenomes from three continents (Europe [53–55], United States [56], and China [51]), as it lacked information on genes specific to the Indian population. The Updated Gene Catalogue (UGC) comprised 11,118,467 genes (an addition of 12.5% genes in the current IGC) with 1,238,571 genes unique to the Indian population.

On this updated gene catalogue, reads from each sample were mapped and the genes present in the Indian population were identified. On average, 54.47% ± 7.84% (average ± SD) of high-quality reads mapped from each sample to UGC and resulted in the identification of 3.8 million genes present in the Indian cohort. Taxonomic assignment and functional annotation were performed for these 3.8 million genes present using 4,097 reference genomes (HMP and NCBI species) and KEGG and eggNOG databases. A total of 2.41 million genes (62.9%) could be successfully assigned a taxonomy at genus level. The remaining genes are expected to be from currently unidentified microbial species. At the functional level, 8,312 KEGG orthologues and 59,303 eggNOG orthologue groups were identified in the updated gene catalogue. Additionally, 24% of the genes which were not mapped to the orthologue groups could be clustered into 649 novel gene families, which did not have any assigned function but were still included in the analysis as novel eggNOG groups.

### Diversity and rarefaction analysis.

Estimation of total gene richness, α-diversity (within-sample diversity), and β-diversity (between-sample diversity) in the set of 60 metagenomic samples was performed by randomized sampling and replacement, and estimates were compared to a different group of samples. Rarefied matrices were obtained by rarefying at 6 million reads per sample. In total, we performed 10 repetitions, and in each of these, we measured richness, α-diversity (by using the Shannon index), and β-diversity (by using Bray-Curtis distance) for each sample. The median values were taken as the respective measurement for each sample. Intersample distances were calculated using the Bray-Curtis distance matrices. The significance of the association with health status was performed using the Wilcoxon rank sum test.

### Phylogenetic assignment of reads.

A total of 4,097 reference microbial genomes were obtained from the Human Microbiome Project (HMP) and National Center for Biotechnology Information (NCBI) on 5 December 2015. The databases were independently indexed into two Bowtie indexes using Bowtie 2 (v2.3.4.1) (57). The metagenomic reads were aligned to the reference microbial genomes using Bowtie 2. The mapped reads from the two indexes were merged by selecting the alignment having the higher identity (≥90% identity). The percent identity was calculated using the formula: % identity = 100 × (matches/total aligned length). The normalized abundance of a microbial genome was calculated by summing the total number of reads aligned to its reference genome, normalized by the genome length and the total number of reads in the data set. For reads showing hits to the two indexed databases with equal identity, each genome was assigned an 0.5 read count. The relative abundance of each genome was calculated by adding the normalized abundance of each genome divided by the total abundance. The Calinski-Harabasz index (CHI) was used to calculate the variance between the clusters compared to the variance within clusters (58). A clade-specific-marker-based taxonomic assignment of reads was also done using the mOTUs (v2) (59) approach and Metaphlan (v2.0) (60).

### Construction of metagenomic species for MGWAS.

The gene cohort and its abundance from 291 samples belonging to India (60), Austria (103), and China (128) were combined and used for determining MGS/CAGs. The Pearson's correlation coefficient (PCC) cutoff of ≥0.9 was used for considering association between genes, and only genes having an abundance of >0 in at least 30 samples were considered for association analysis. Furthermore, the genes for which ≥90% abundance was obtained from a single sample were discarded. To determine the taxonomic origin of each MGS/CAG (metagenomic cluster), all the genes were aligned against reference microbial genomes of 4,097 genomes from HMP and NCBI at nucleotide level using BLASTN. The alignment hits were filtered using an E value of ≤10^−6^ and alignment coverage of ≥80% of the gene length, and 2,687,688 genes showed alignments against the reference genomes. The remaining genes were aligned against the UNIREF database (UniRef50) at protein sequences (61). The multiple best hits with equal identity and scores were further assigned taxonomy based on the lowest common ancestor (LCA) method. The genes were finally assigned to taxa based on comprehensive parameters of sequence similarity across phylogenetic ranks as described earlier (62). The identity threshold of ≥95% was used for assignment up to species level, ≥85% identity threshold was used for assignment up to genus level, and ≥65% identity was used for phylum-level assignment using BLASTN. The taxonomic assignments of MGS/CAGs were performed with the criterion that ≥50% of genes in each MGS should map to the same lowest phylogenetic group. So, if a particular species is assigned ≥50% genes out of the total, the assignment will be carried out at species level rather than at the level of genus or higher orders. The relative abundance of MGS/CAGs in each sample was estimated by using relative abundance values of all genes from that MGS/CAG. A Poisson distribution was fitted to the relative abundance values of the data. The mean estimated from Poisson distribution was assigned as the relative abundance of that MGS. The profiles of MGS/CAGs were generated and used for further analysis. The MGS/CAGs associated with CRC in the Indian population were scored using log odds ratio, and *P* values were calculated using the Wilcoxon rank sum test between CRC and healthy individuals. The Wilcoxon rank sum test was adjusted for multiple comparisons using false-discovery rate (FDR) adjustment. The MGS having *P* values of <0.05 and log odds ratio of >2 (CRC) or ≤2 (healthy) were considered enriched in CRC or healthy groups, respectively.

### Fecal metabolomic sample preparation and derivatization.

In order to identify the metabolic potential of microbes, metabolomics profiling of a subset of individuals (*n* = 18; CRC patients = 9, healthy = 9) was performed. Lyophilized fecal samples were used to achieve better metabolite coverage, as described previously (12). Metabolites were extracted from 80 mg of lyophilized samples in 1 ml of ice-cold methanol-water (8:2) by bead beating for 30 cycles (each cycle included 30 s of beating at 2,500 rpm and 1 min of standing at 4°C). The samples were then sonicated for 30 min in a probe-based sonicator (Branson digital Sonifier, model 102 C with double-step microtip) at 4°C followed by 2 min of vortexing. The supernatant was extracted by centrifugation at 18,000 × *g* for 15 min at 4°C and dried at 50°C under a gentle stream of nitrogen gas. To remove the residual water molecules from the samples, 100 μl of toluene was added to the dry residue and evaporated completely at 50°C under nitrogen gas. The extracted metabolites were first derivatized with 50 μl of methoxyamine hydrochloride (MOX) in pyridine (20 mg/ml) at 60°C for 2 h, and the second derivatization was performed with 100 μl of *N*-methyl-*N*-(trimethylsilyl)trifluoroacetamide (MSTFA) in 1% trimethylchlorosilane (TMCS) at 60°C for 45 min to form trimethylsilyl (TMS) derivatives. Finally, 150 μl of the TMS derivatives was transferred into a GC glass vial insert and subjected to GC-time of flight MS (TOFMS) analysis.

### GC-MS analysis.

GC-MS was performed on an Agilent 7890A gas chromatograph with a 5975 C MS system. An HP-5 (25-m by 320-μm by 0.25-μm-inside-diameter [i.d.]) fused silica capillary column (Agilent J&W Scientific, Folsom, CA) was used with the open split interface. The injector, transfer line, and ion source temperatures were maintained at 220, 220, and 250°C, respectively. Oven temperature was programmed at 70°C for 0.2 min and increased at 10°C/min to 270°C, where it was sustained for 5 min, and further increased at 40°C/min to 310°C, where it was held for 11 min. The MS was operated in the electron impact ionization mode at 70 eV. Mass data were acquired in full scan mode from *m/z* 40 to 600 with an acquisition rate of 20 spectra per second. To detect retention time shifts and enable Kovats retention index (RI) calculation, a standard alkane series mixture (C_10_ to C_40_) was injected periodically during the sample analysis. RIs are relative retention times normalized to *n*-alkanes eluted adjacently. The injector port temperature was held at 250°C, and the helium gas flow rate was set to 1 ml/min at an initial oven temperature of 50°C. The oven temperature was increased at 10°C/min to 310°C for 11 min, and mass data were acquired in full scan mode from *m/z* 40 to 600 with an acquisition rate of 20 spectra per second.

### Metabolomic analysis.

The raw peaks were processed for peak identification and alignment using the XCMS package in R. Initially, prior to alignment the parameters used for peak picking and retention time correction were optimized using the IPO package in R. The package using iterative modes and a range of values optimizes the best parameter settings for the GC-MS experiment. Using the centwave algorithm, the peaks were detected from 18 samples and were further corrected for retention time corrections using a “binwidth” of 1. The parameters optimized for preprocessing of the peaks from the GC-MS experiment are as follows: minimum peak width = 3, maximum peak width = 18.75, parts per million [ppm] = 575, difference in *m/z* values = 0.004588, signal-to-noise ratio [S/N] threshold = 10, bin width size = 1. The peaks were annotated using NIST/Massbank databases.

The most prominent features of the GC-MS data which could be annotated were used for differential analysis. In total, 234 features that were well annotated and showed prominent peaks were used. The differential analysis of peaks was performed using the MetaboDiff package in R. The concentrations of fecal metabolites in CRC and healthy subjects were quantified from peak intensities and were further normalized.

### Meta-analysis.

In order to define a non-reference-based approach of assigning genetic markers to a particular condition (CRC in this case), we used the MGWAS approach, which uses correlations among genes to generate clusters of genes. Various methods have been described for clustering genes in previous studies (51, 63). We here used a recently published method of determining clusters of genes using MGS-canopy-based correlations (55). The approach uses a matrix of gene abundance across samples and the correlation coefficient cutoffs used to identify fine-grained clusters of genes belonging to single species or closely related species.

The gene cohort and its abundance from 291 samples belonging to India (60), Austria (103), and China (129) were combined and used for determining MGS/CAGs. The PCC cutoff of ≥0.9 was used for considering association between genes, and only genes having an abundance of >0 in at least 30 samples were considered for association analysis. Furthermore, the genes for which ≥90% abundance was obtained from a single sample were discarded. To determine the taxonomic origin of each MGS/CAG (metagenomic cluster), all the genes were aligned against reference microbial genomes of 4,097 genomes from HMP and NCBI at nucleotide level using BLASTN. The alignment hits were filtered using an E value of ≤10^−6^ and alignment coverage of ≥80% of the gene length, and 2,687,688 genes showed alignments against the reference genomes. The remaining genes were aligned against the UNIREF database (UniRef50) at protein sequences (61). The multiple best hits with equal identity and scores were further assigned taxonomy based on the lowest common ancestor (LCA) method. The genes were finally assigned to taxa based on comprehensive parameters of sequence similarity across phylogenetic ranks as described earlier (62). The identity threshold of ≥95% was used for assignment up to species level, an ≥85% identity threshold was used for assignment up to genus level, and ≥65% identity was used for phylum-level assignment using BLASTN. The taxonomic assignments of MGS/CAGs were performed with the criteria that ≥50% of genes in each MGS should map to the same lowest phylogenetic group. So, if a particular species is assigned ≥50% genes out of the total, the assignment will be carried out at species level rather than at the level of genus or higher orders. The relative abundance of MGS/CAGs in each sample was estimated by using relative abundance values of all genes from that MGS/CAG. A Poisson distribution was fitted to the relative abundance values of the data. The mean estimated from Poisson distribution was assigned as the relative abundance of that MGS. The profiles of MGS/CAGs were generated and used for further analysis.

### Global comparative analysis.

The MGS/CAG abundance table was rarefied for 100 times at a lowest sequencing depth of 220,000 sequences/sample. The average of these rarefied counts from 100 iterations was calculated and used for further analysis. The principal-component analysis was performed on the MGS abundance table, and the components were correlated with covariates (country and status) using polyserial correlation. PCA with polyserial correlations showed countrywide variations as a major factor correlated with PC1.

Since variations between countries were higher than those between CRC patients, we performed multivariate analysis distance-based redundancy analysis (db-RDA). Canberra distances were selected based on the highest rank index for performing db-RDA. The metainformation used was the status (CRC/healthy) and country/study from which the data set came (India, China, and Austria). The Bray-Curtis distances were used for constrained ordinations and were constrained for country and status. The function “capscale” was used for performing ordinations with country and status separately, and the ordinations were plotted on a PCA plot.

### Identifying global taxonomic patterns.

In order to identify global CRC MGS/CAG, we performed univariate testing for differential abundance of MGS/CAG. Since countries and populations are stratified and have variations between them, we controlled for population variations by applying block. The independence test was performed using ytrafo = rank for Wilcoxon rank sum test and teststat = “scalar” using the COIN package in R. The population variations were controlled, and differential abundances of MGS/CAGs were calculated.

### Gene biomarker identification.

For gene biomarker identification, the 60 samples were divided into two cohorts: cohort A and cohort B. Cohort A comprised 48 samples and cohort B comprised 12 samples, by randomly selecting from each location and health status. Using the samples of cohort A, a group of genes that were highly correlated with each other (Pearson correlation ρ > 0.9) were identified, and the longest gene from each correlated group was used to construct a statistically nonredundant set. Further, we used the “CfsSubsetEval” method from Weka to identify a subset of genes that are highly correlated with the health status while having low intercorrelation with each other. The genes from this subset were further validated using the Boruta algorithm, which uses Random Forest to perform a top-down search for relevant features by comparing original attribute importance with importance achievable at random and eliminates irrelevant features to stabilize the test. To test the accuracy of these markers, a Random Forest model was constructed using these genes and was used for making the prediction on samples from cohort B.

### CRC index.

To compare the performances of markers, we computed a CRC index, as defined by Yu et al. (6), for each of the individuals on the basis of 33 gene markers identified using the methodology mentioned above.

### Supervised learning.

Predictive models were built using supervised machine learning algorithm Random Forest (RF). The models were optimized using 10,000 trees and default settings of mtry (number for variables used to build the model). The mean 3-fold cross-validation error rates were calculated for each of the binary trees and the ensemble of trees. The mean decrease in accuracy, which is the increase in error rates on leaving the variable out, was calculated for each prediction and tree and was used to estimate the importance score. The variables showing a higher mean decrease in accuracy of prediction were considered important for the segregation of the data sets into groups based on the categorical variable.

### Network plot.

In order to derive associations between microbial markers, a species cooccurrence network was generated from pairwise correlations using sparCC, which takes into account the compositional data and estimates the correlations between species. The species associations with correlation coefficients of >0.3 were considered for construction of networks and inferring associations among species.

### Statistical analysis.

All the statistical comparisons between groups were performed using a nonparametric Wilcoxon rank sum test with FDR-adjusted *P* values to control for multiple comparisons. The correlations between two variables and the correlations within the variable were calculated using Spearman’s correlation coefficient with adjusted *P* values. The correlations between categorical and numeric variables were performed using polyserial correlation/biserial correlations. To identify the enrichment of enzymes/species associated with a host, odds ratio was used as a measure of the enrichment of an enzyme in a host. The odds ratio was calculated as OR (*k*) = [∑*_S_*_ = LOC1_ *A_Sk_*/∑*_S_*_ = LOC1_ (∑*_i_*_≠_ *_k_ A_Si_*)]/[∑*_S_*_ = LOC2_ *A_Sk_*/∑*_S_*_ = LOC2_ (∑*_i_*_≠_ *_k_ A_Si_*)], where *A_Sk_* denotes abundance of enzyme *k* in sample *S*. Apart from that, the Reporter features algorithm was used for gene-set analysis of significant pathways associated with different groups of samples. The algorithm takes the adjusted *P* values and fold changes (log odds ratio) as input for each KO. The gene statistic is calculated based on the significant association of KO and its direction of change through which the pathway is scored by calculating the global *P* value. All the graphs and plots were generated using the ggplot2 package in R.

### Ethics approval and consent to participate.

The recruitment of volunteers, sample collection, and other study-related procedures were carried out by following the guidelines and protocols approved by the Institute Ethics Committee of the Indian Institute of Science Education and Research (IISER), Bhopal, India. A written informed consent was obtained from all the subjects prior to any study-related procedures.

### Availability of data and material.

The data sets generated and/or analyzed during the current study are available in the NCBI BioProject database under project numbers PRJNA531273 and PRJNA397112.
