# Supplementary figures and images for: Association of Flavonifractor plautii, a Flavonoid-Degrading Bacterium, with the Gut Microbiome of Colorectal Cancer Patients in India
Source: mSystems. 2019 Nov 12;4(6):e00438-19. doi: 10.1128/mSystems.00438-19 (PMC7407896; doi:10.1128/mSystems.00438-19)

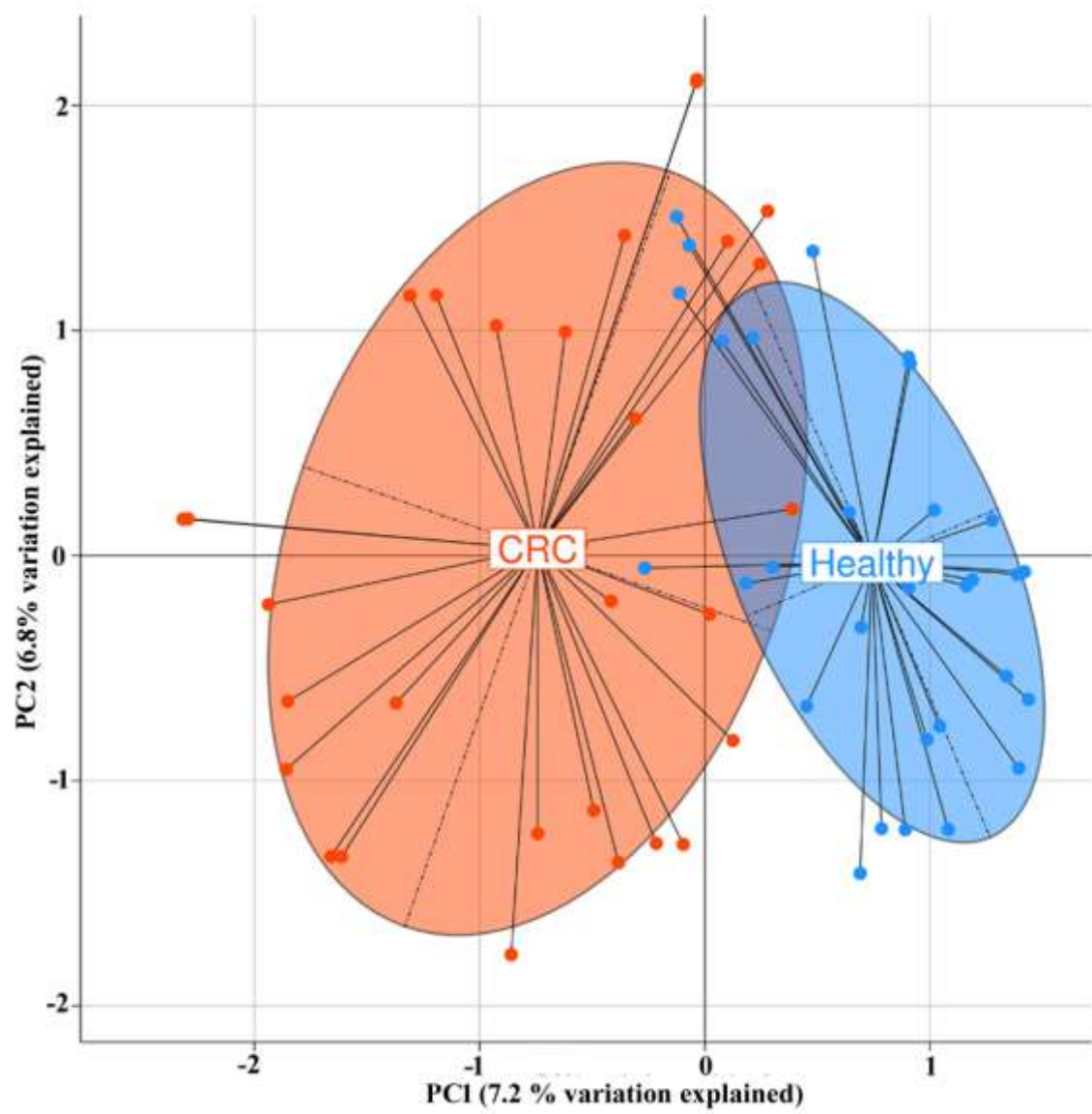

Supplement: FIG S1 [file mSystems.00438-19-sf001.pdf]

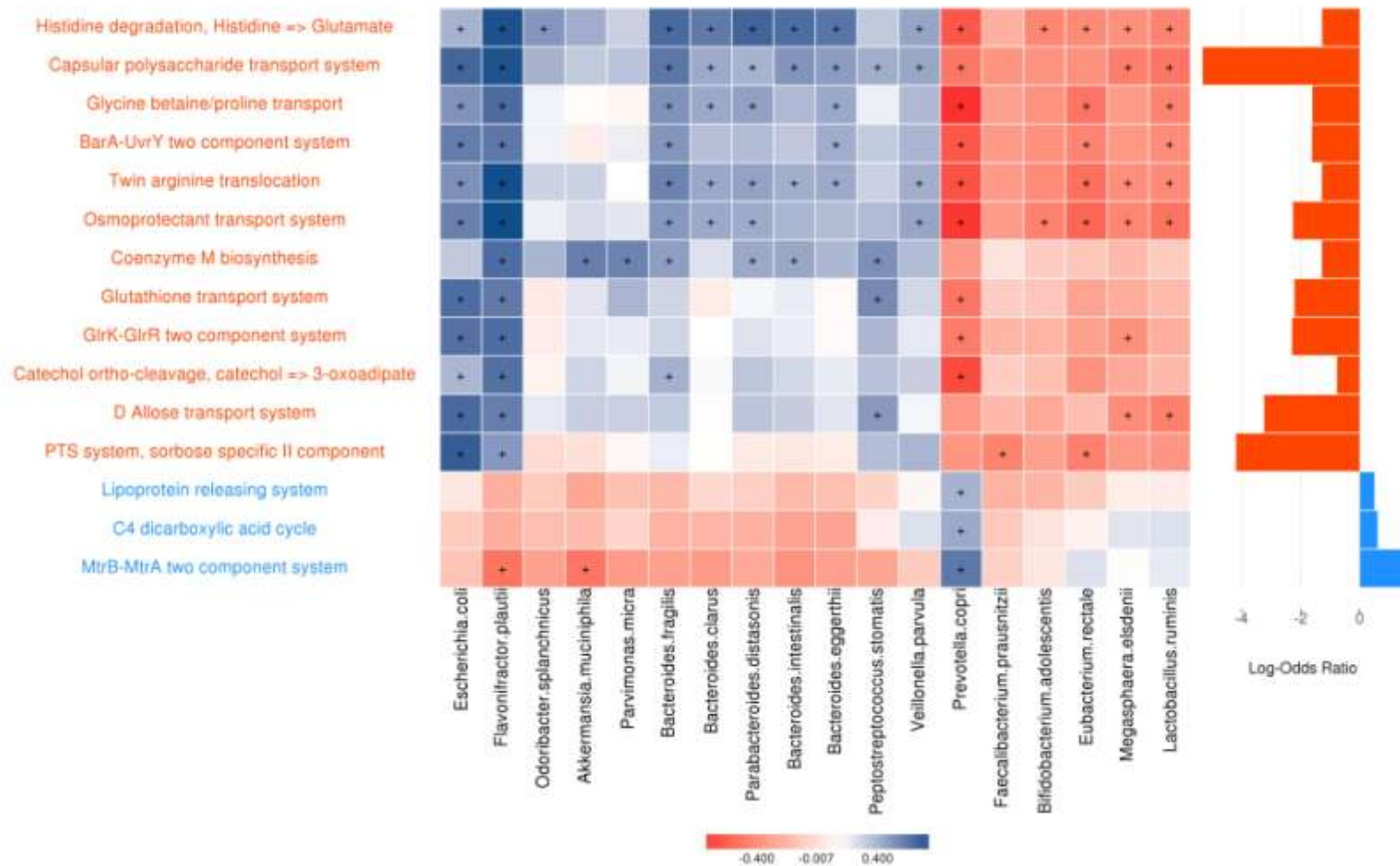

Supplement: FIG S2 [file mSystems.00438-19-sf002.pdf]

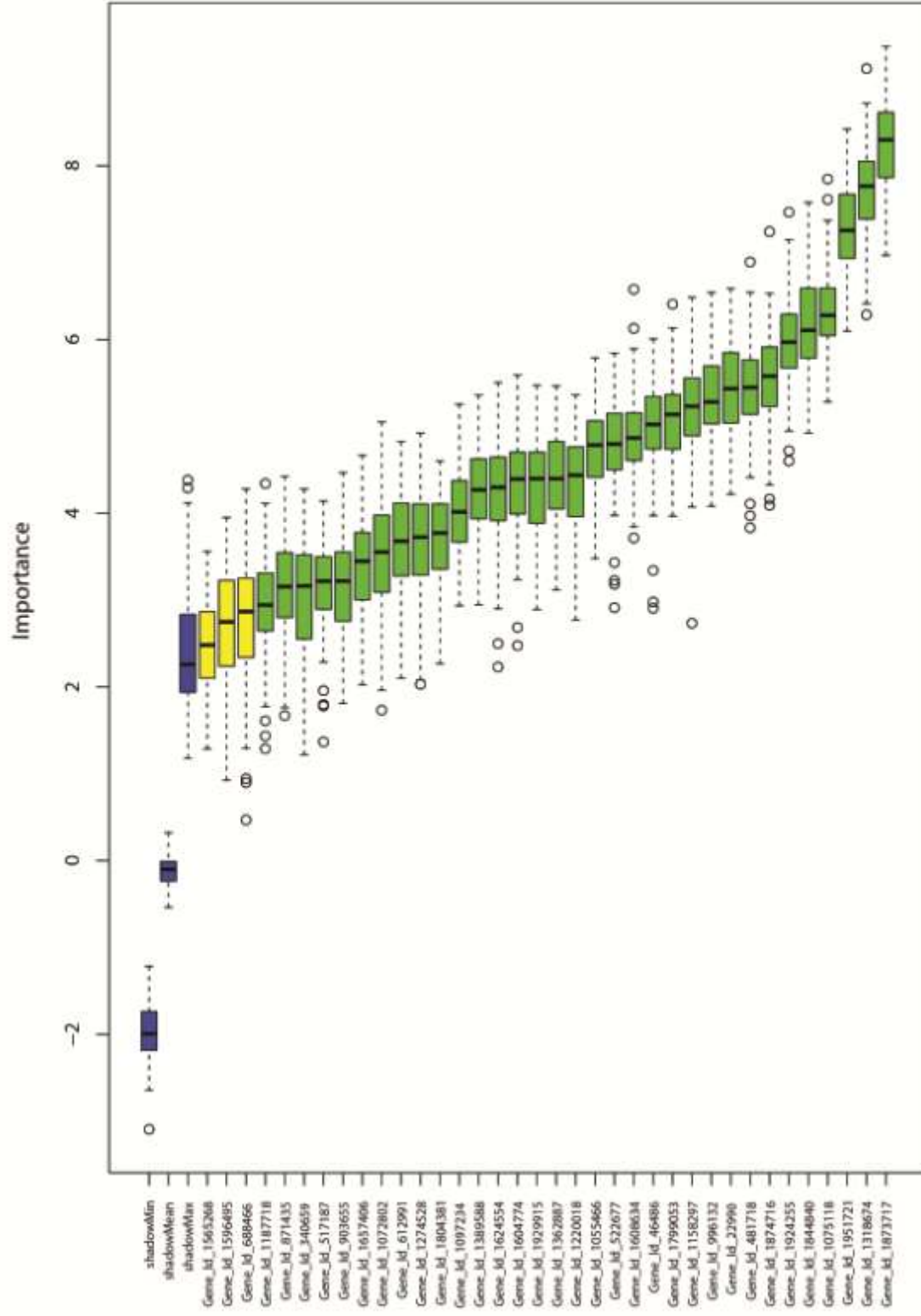

Supplement: FIG S3 [file mSystems.00438-19-sf003.pdf]
