# Supplementary material for: Association of Flavonifractor plautii, a Flavonoid-Degrading Bacterium, with the Gut Microbiome of Colorectal Cancer Patients in India
Source: mSystems. 2019 Nov 12;4(6):e00438-19. doi: 10.1128/mSystems.00438-19 (PMC7407896; doi:10.1128/mSystems.00438-19)
Supplement: TABLE S2 [file mSystems.00438-19-st002.docx]

**Table S2.** Polyserial correlations of the covariates with the 10 principal components, calculated using microbial gene profiles from 60 samples using 1.9 million genes.

| Axis | Covariate | Z-score | P-value | Adjusted P-value |
| --- | --- | --- | --- | --- |
| PC1 | Status | 10.4664 | 1.23E-25 | **< 0.000001** |
| PC1 | Age | -2.5567 | 0.0106 | 0.0170 |
| PC1 | Gender | -2.4052 | 0.0162 | 0.0200 |
| PC1 | BMI | 2.5922 | 0.0095 | 0.0170 |
| PC1 | Location | 1.8091 | 0.0704 | 0.0704 |
| PC2 | Status | -0.2348 | 0.8144 | 0.8140 |
| PC2 | Age | -0.3427 | 0.7318 | 0.8140 |
| PC2 | Gender | -0.5563 | 0.5780 | 0.8140 |
| PC2 | BMI | 1.0990 | 0.2718 | 0.6790 |
| PC2 | Location | 4.4402 | 8.99E-06 | **0.000044** |
| PC3 | Status | -2.6053 | 0.0092 | 0.0450 |
| PC3 | Age | 2.2211 | 0.0263 | 0.0650 |
| PC3 | Gender | 0.2477 | 0.8044 | 0.8040 |
| PC3 | BMI | -1.6476 | 0.0994 | 0.1240 |
| PC3 | Location | 1.9166 | 0.0553 | 0.0920 |
| PC4 | Status | -1.7792 | 0.0752 | 0.3513 |
| PC4 | Age | 0.4783 | 0.6325 | 0.6324 |
| PC4 | Gender | 1.0580 | 0.2900 | 0.4834 |
| PC4 | BMI | -0.6604 | 0.5090 | 0.6325 |
| PC4 | Location | -1.4739 | 0.1405 | 0.3513 |
| PC5 | Status | 0.3391 | 0.7345 | 0.9510 |
| PC5 | Age | -0.0610 | 0.9514 | 0.9510 |
| PC5 | Gender | 0.3217 | 0.7477 | 0.9510 |
| PC5 | BMI | 0.6539 | 0.5132 | 0.9510 |
| PC5 | Location | 0.2579 | 0.7964 | 0.9510 |
| PC6 | Status | -0.9701 | 0.3320 | 0.4140 |
| PC6 | Age | 0.7636 | 0.4451 | 0.4450 |
| PC6 | Gender | 2.1889 | 0.0286 | 0.1350 |
| PC6 | BMI | -1.9257 | 0.0541 | 0.1350 |
| PC6 | Location | 1.1806 | 0.2378 | 0.3960 |
| PC7 | Status | 0.9589 | 0.3376 | 0.9050 |
| PC7 | Age | -0.2568 | 0.7973 | 0.9050 |
| PC7 | Gender | 0.3900 | 0.6965 | 0.9050 |
| PC7 | BMI | 0.7102 | 0.4776 | 0.9050 |
| PC7 | Location | 0.1193 | 0.9050 | 0.9050 |
| PC8 | Status | 0.3495 | 0.7267 | 0.8260 |
| PC8 | Age | 1.8081 | 0.0706 | 0.1760 |
| PC8 | Gender | 0.2197 | 0.8261 | 0.8260 |
| PC8 | BMI | -1.4227 | 0.1548 | 0.2580 |
| PC8 | Location | 1.8571 | 0.0633 | 0.1760 |
| PC9 | Age | 1.0643 | 0.2872 | 0.7690 |
| PC9 | Gender | 0.7596 | 0.4475 | 0.7690 |
| PC9 | BMI | -0.6543 | 0.5129 | 0.7690 |
| PC9 | Location | -0.5018 | 0.6158 | 0.7690 |
| PC10 | Status | -0.6317 | 0.5276 | 0.6594 |
| PC10 | Age | -0.2011 | 0.8406 | 0.8406 |
| PC10 | Gender | -1.5811 | 0.1139 | 0.5693 |
| PC10 | BMI | 0.6402 | 0.5221 | 0.6594 |
| PC10 | Location | -0.9732 | 0.3304 | 0.6594 |
