# Supplementary material for: Association of Flavonifractor plautii, a Flavonoid-Degrading Bacterium, with the Gut Microbiome of Colorectal Cancer Patients in India
Source: mSystems. 2019 Nov 12;4(6):e00438-19. doi: 10.1128/mSystems.00438-19 (PMC7407896; doi:10.1128/mSystems.00438-19)
Supplement: TABLE S4 [file mSystems.00438-19-st004.docx]

**Table S4.** PERMANOVA analysis on taxonomic species identified using four different strategies to test the impact of health status, sample location and clinical parameters on the gut microbiota with q < 0.01.

| **Parameter** | **Df** | **MGS Species** | | | | |
| --- | --- | --- | --- | --- | --- | --- |
|  |  | **S.Sq** | **F.Model** | **R2** | **Pr (> F)** | **q-value** |
| **Health status** | 1 | 2.3860 | 9.7400 | 0.1300 | 0.0009 | **0.0040** |
| **Age** | 1 | 0.4388 | 1.7913 | 0.0200 | 0.0300 | 0.0900 |
| **Gender** | 1 | 0.2981 | 1.2167 | 0.0200 | 0.1980 | 0.3560 |
| **BMI** | 1 | 0.2638 | 1.0769 | 0.0100 | 0.3500 | 0.5220 |
| **Location** | 1 | 0.8870 | 3.6240 | 0.0400 | 0.0009 | **0.0040** |
| **TNM Staging** | 1 | 0.3641 | 1.4860 | 0.0200 | 0.0650 | 0.1460 |
| **Histopathology** | 1 | 0.2130 | 0.8730 | 0.0100 | 0.6000 | 0.6750 |
| **Localization** | 1 | 0.2470 | 1.0090 | 0.1000 | 0.4060 | 0.5220 |
|  |  | **Metaphlan Species** | | | | |
| **Health status** | 1 | 2.9800 | 10.5100 | 0.1300 | 0.0009 | **0.0040** |
| **Age** | 1 | 0.4120 | 1.4500 | 0.0100 | 0.0970 | 0.2630 |
| **Gender** | 1 | 0.3100 | 1.0900 | 0.1400 | 0.3250 | 0.4240 |
| **BMI** | 1 | 0.3440 | 1.2120 | 0.0100 | 0.1970 | 0.3540 |
| **Location** | 1 | 0.9431 | 3.3200 | 0.0400 | 0.0009 | **0.0040** |
| **TNM Staging** | 1 | 0.3912 | 1.3770 | 0.0100 | 0.1170 | 0.2630 |
| **Histopathology** | 1 | 0.2870 | 1.0120 | 0.0100 | 0.4240 | 0.4240 |
| **Localization** | 1 | 0.2830 | 0.9900 | 0.0100 | 0.4180 | 0.4240 |
|  |  | **mOTU Species** | | | | |
| **Health status** | 1 | 2.8430 | 11.1900 | 0.1000 | 0.0009 | **0.0040** |
| **Age** | 1 | 0.2164 | 0.8522 | 0.0100 | 0.6040 | 0.6040 |
| **Gender** | 1 | 0.2927 | 1.1500 | 0.0100 | 0.2710 | 0.4870 |
| **BMI** | 1 | 0.2520 | 0.9940 | 0.0100 | 0.4380 | 0.5140 |
| **Location** | 1 | 0.8700 | 3.4270 | 0.0400 | 0.0009 | **0.0040** |
| **TNM Staging** | 1 | 0.3900 | 1.5390 | 0.0100 | 0.0760 | 0.2280 |
| **Histopathology** | 1 | 0.2500 | 0.9850 | 0.0100 | 0.4570 | 0.5140 |
| **Localization** | 1 | 0.3060 | 1.2070 | 0.0100 | 0.2250 | 0.4870 |
|  |  | **HMP-NCBI Species** | | | | |
| **Health status** | 1 | 2.4331 | 12.6733 | 0.1742 | 0.0009 | **0.0079** |
| **Age** | 1 | 0.2349 | 1.2236 | 0.0166 | 0.2367 | 0.4224 |
| **Gender** | 1 | 0.2215 | 1.1539 | 0.0157 | 0.2727 | 0.4224 |
| **BMI** | 1 | 0.2012 | 1.0481 | 0.0142 | 0.3696 | 0.4224 |
| **Location** | 1 | 0.6671 | 3.4747 | 0.0472 | 0.0039 | **0.0159** |
| **TNM Staging** | 1 | 0.2183 | 1.1372 | 0.0154 | 0.2937 | 0.4224 |
| **Histopathology** | 1 | 0.1292 | 0.6731 | 0.0991 | 0.8191 | 0.8191 |
| **Localization** | 1 | 0.2092 | 1.0894 | 0.0148 | 0.3296 | 0.4224 |
